# The Fifty per Cent. Resolution of “Examiners”

**Published:** 1903-11

**Authors:** 


### THE FIFTY PER CENT. RESOLUTION OF "EXAMINERS."

THERE has been so much misunderstanding in regard to what the National Association of Dental Examiners decided upon at Asheville, N. C., that the secretary, Dr. C. A. Meeker, was requested to furnish a correct copy of the resolution adopted. This was very courteously given and it is therefore appended, as furnished from that authoritative source.

"*Ordered,* That the Committee on Colleges be and they are hereby ordered and directed to revise the list of recommended colleges of this Association; and be it further ordered and directed that the Committee on Colleges obtain from the various State Boards a statement showing the number examined, passed, and rejected from each college, and that when it appears that fifty per cent. of the graduate students from any college fail to pass in States where examination is necessary for registration that the Committee on Colleges shall report such facts to this Association for such action as may seem fit and proper."

Any one reading this intelligently will see that the editorial in September number entitled "A Step Backward" was not very far wrong in its conclusions. The National Association of Dental Examiners cannot perfect a list of colleges, under this rule, until the examinations are finally closed in June of 1904. The resolution does not require any fixed period for the course, consequently this is left to the judgment of the State boards. It is impossible to discover in this resolution anything, but the State law, to prevent any board from examining students from colleges that have not adopted the four years' course ordered by the Association of Faculties. If the National Association of Dental Examiners has any power to control State boards, it has practically antagonized the dental colleges in their efforts to increase the length of term, and it is impossible to see why a State board could not examine students of a college having a one-year session. Under some State laws this, of course, could not be done, but the majority of said laws simply require a "reputable college." The word reputable is very elastic, and could apply to a college of one year as well as one of four years, it all depending on the decision of the State board.

The members of the National Association of Dental Examiners who have criticised sharply this journal for said September editorial evidently failed to understand the drift of the resolution to which they gave their support at the Asheville meeting.

